# Expression of the SARS-CoV-2 cell receptor gene *ACE2* in a wide variety of human tissues

**DOI:** 10.1186/s40249-020-00662-x

**Published:** 2020-04-28

**Authors:** Meng-Yuan Li, Lin Li, Yue Zhang, Xiao-Sheng Wang

**Affiliations:** 1 Biomedical Informatics Research Lab, School of Basic Medicine and Clinical Pharmacy, China Pharmaceutical University, Nanjing, 211198 China; 2 Big Data Research Institute, China Pharmaceutical University, Nanjing, 211198 China; 3 Pinghu hospital of Shenzhen University, Shenzhen, 440307 China; 4 Futian Hospital for Rheumatic Diseases, Shenzhen, 518000 China; 5 Department of Rheumatology and Immunology, The First Clinical College of Harbin Medical University, Harbin, 150001 China

**Keywords:** SARS-CoV-2, COVID-19, SARS-CoV-2 cell receptor, Angiotensin-converting enzyme 2, Gene expression, SARS-CoV-2 pandemic, Immune signatures

## Abstract

**Background:**

Since its discovery in December 2019, severe acute respiratory syndrome coronavirus 2 (SARS-CoV-2) has infected more than 2 180 000 people worldwide and has caused more than 150 000 deaths as of April 16, 2020. SARS-CoV-2, which is the virus causing coronavirus disease 2019 (COVID-19), uses the angiotensin-converting enzyme 2 (ACE2) as a cell receptor to invade human cells. Thus, ACE2 is the key to understanding the mechanism of SARS-CoV-2 infection. This study is to investigate the ACE2 expression in various human tissues in order to provide insights into the mechanism of SARS-CoV-2 infection.

**Methods:**

We compared *ACE2* expression levels across 31 normal human tissues between males and females and between younger (ages ≤ 49 years) and older (ages > 49 years) persons using two-sided Student’s *t* test. We also investigated the correlations between *ACE2* expression and immune signatures in various tissues using Pearson’s correlation test.

**Results:**

*ACE2* expression levels were the highest in the small intestine, testis, kidneys, heart, thyroid, and adipose tissue, and were the lowest in the blood, spleen, bone marrow, brain, blood vessels, and muscle. *ACE2* showed medium expression levels in the lungs, colon, liver, bladder, and adrenal gland.* ACE2* was not differentially expressed between males and females or between younger and older persons in any tissue. In the skin, digestive system, brain, and blood vessels, *ACE2* expression levels were positively associated with immune signatures in both males and females. In the thyroid and lungs,* ACE2* expression levels were positively and negatively associated with immune signatures in males and females, respectively, and in the lungs they had a positive and a negative correlation in the older and younger groups, respectively.

**Conclusions:**

Our data indicate that SARS-CoV-2 may infect other tissues aside from the lungs and infect persons with different sexes, ages, and races equally. The different host immune responses to SARS-CoV-2 infection may partially explain why males and females, young and old persons infected with this virus have markedly distinct disease severity. This study provides new insights into the role of ACE2 in the SARS-CoV-2 pandemic.

## Background

The outbreak of severe acute respiratory syndrome coronavirus 2 (SARS-CoV-2) is rapidly spreading across the world and have caused a global health emergency [1]. Patients infected with SARS-CoV-2 have mainly displayed pneumonia-associated symptoms, including fever, cough, shortness of breath, sputum production, and myalgia or fatigue [2, 3], indicating that SARS-CoV-2 primarily infects the respiratory tract and causes acute respiratory disease. However, SARS-CoV-2 infection may result in symptoms of diseases associated with other tissues, such as digestive (diarrhea, poor appetite, nausea, and vomiting), nervous (confusion and headache), and cardiovascular (palmus, chest distress, and cardiac injury) systems [2, 3]. In addition, some studies have indicated that person-to-person transmission of SARS-CoV-2 can occur by routes outside of the respiratory tract [4]. A study of 99 patients infected with SARS-CoV-2 showed that females were less susceptible to infection than males, and older males with comorbidities were more likely to be infected with SARS-CoV-2 [3]. Like SARS-related coronavirus (SARS-CoV) [5], SARS-CoV-2 uses the angiotensin-converting enzyme 2 (ACE2) as a host cell receptor [6–8]. A recent study uncovered that *ACE2*-expressing lung cells were more abundant in Asian males [9], potentially explaining the elevated susceptibility of males to SARS-CoV-2 infection. Nevertheless, the findings from that study are not sufficiently convincing due to a small number of samples being analyzed.

In this study, we analyzed the expression of *ACE2* in various normal human tissues using the datasets from the Genotype-Tissue Expression (GTEx) project [10] and The Cancer Genome Atlas (TCGA) program (https://portal.gdc.cancer.gov/). We compared *ACE*2 expression levels across 31 human tissues, between males and females, and between younger and older persons in these individual tissues. Furthermore, we analyzed the correlations between *ACE2* expression levels and immune signature enrichment levels in individual tissues.

## Methods

### Datasets

We downloaded the GTEx RNA-Seq gene expression profiling datasets (RSEM normalized) for 31 human normal tissues from the UCSC Xena project (https://xenabrowser.net/datapages/). We downloaded the TCGA RNA-Seq gene expression profiling datasets (RSEM normalized) for 12 human normal tissues from the Genomic Data Commons Data Portal (https://portal.gdc.cancer.gov/). All gene expression values were added to 0.001 and then log2-transformed before analysis.

### Evaluation of the immune signature enrichment levels in tissue

We defined the enrichment level of an immune signature in tissue as the mean expression level of marker genes of the immune signature in the tissue. We analyzed four immune signatures, including CD8+ T cells, interferon response, B cells, and natural killer (NK) cells. The CD8+ T cell marker genes included *CD2*, *CD247*, *CD28*, *CD3D*, *CD3E*, *CD3G*, *CD8A*,* ICAM1*, *ITGAL*,* ITGB2*, *PTPRC*, and *THY1* [11]. The interferon response marker genes included* IFIT1*, *IFIT2*, *IFIT3*, *IRF7*,* ISG20*, *MX1*, *MX2*, *RSAD2*, *TNFSF10*, *GPR146*, *SELP*, and *AHR* [12]. The B cell marker genes included *BACH2*, *BANK1*,* BLK*, *BTLA*, *CD79A*, *CD79B*, *FCRL1*, *FCRL3*, *HVCN1*, and *RALGPS2* [12]. The NK cell marker genes included *KLRC1* and *KLRF1* [12].

### Statistical analysis

We used Pearson’s correlation test to calculate the correlations between *ACE2* expression levels and immune signature enrichment levels in individual tissues. We used a Student’s *t* test (two-sided) to compare *ACE2* expression levels between males and females, between younger (ages ≤ 49 years) and older (ages > 49 years) persons, and between Asian and non-Asian races in individual tissues. The adjusted *P* value estimated by the Benjamini and Hochberg method [13] was used to adjust for multiple tests.

## Results

### *ACE2* expression in various human tissues

**Fig. 1:**
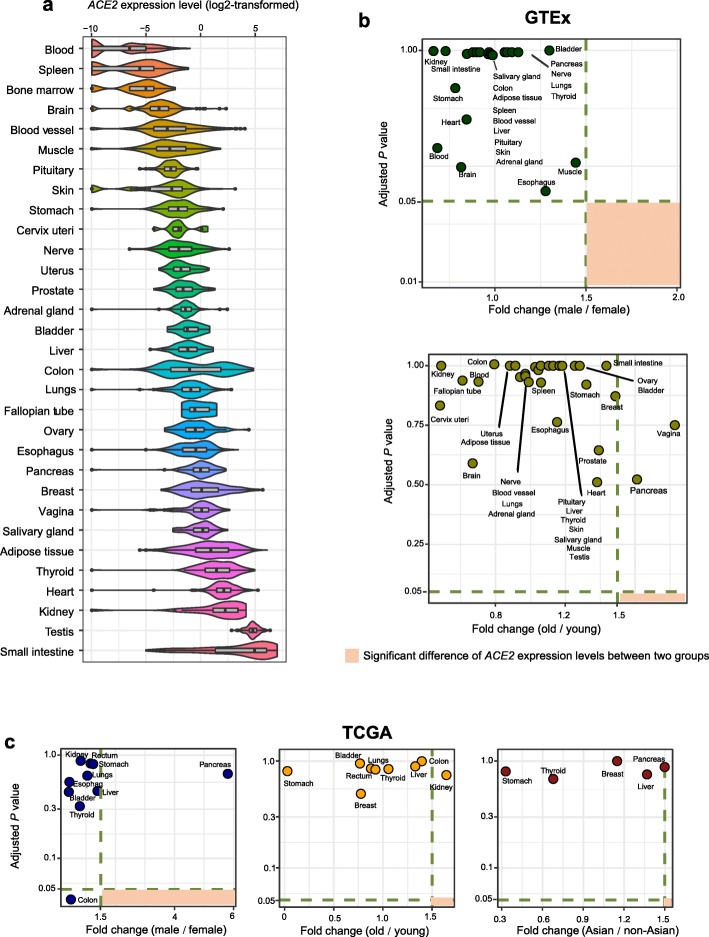
*ACE2* expression in various human tissues. **a** Comparison of *ACE2* expression levels across 31 human tissues in GTEx [10]. Comparison of* ACE2* expression levels between males and females and between younger (ages ≤ 49 years) and older (ages > 49 years) groups in individual human tissues in GTEx (**b**) and TCGA (**c**). In TCGA, a comparison of *ACE2* expression levels between Asian and non-Asian races in five normal human tissues was also performed (**c**). Two-sided Student’s *t* test was used in (**b**, **c**). The adjusted *P* value was calculated by the Benjamini and Hochberg method [13]. ACE2: Angiotensin-converting enzyme 2; GTEx: Genotype-Tissue Expression; TCGA: The Cancer Genome Atlas

Among the 31 GTEx human tissues, the small intestine, testis, kidneys, heart, thyroid, and adipose tissue had the highest *ACE2* expression levels, while blood, spleen, bone marrow, brain, blood vessels, and muscle had the lowest *ACE2* expression levels (Fig. 1a). In the lungs, colon, liver, bladder, and adrenal gland, *ACE2* showed medium expression levels (Fig. 1a). These results suggest that *ACE2* is expressed in a wide variety of human tissues in addition to the lungs.

Furthermore, the Human Protein Atlas (HPA) database (http://www.proteinatlas.org/) showed that the ACE2 protein had relatively high expression levels in the duodenum, small intestine, gallbladder, kidneys, testis, seminal vesicle, colon, rectum, and adrenal gland. The HPA database also showed that the gastrointestinal tract (duodenum, small intestine, colon, and rectum), kidney, gallbladder, and male tissues (testis and seminal vesicle) had high expression levels of both ACE2 gene and protein.

Taken together, these data indicate that: i) SARS-CoV-2 may infect other human tissues in addition to lungs, and ii) males may be more susceptible to SARS-CoV-2 infection than females.

We further compared *ACE2* expression levels between males and females in 22 individual human tissues using the GTEx datasets and found no statistically significant difference between males and females in any tissue using a threshold of adjusted *P* value < 0.05 and fold change > 1.5 (Fig. 1b). We also compared *ACE2* expression levels between younger (ages ≤ 49 years) and older (ages > 49 years) populations and did not observe a statistically significant difference between the groups in any tissue with a threshold of adjusted *P* value < 0.05 and fold change > 1.5 (Fig. 1b). Likewise, in TCGA datasets, *ACE2* was not differentially expressed between males and females or between younger and older groups in any tissue (Fig. 1c), consistent with the results in GTEx. In TCGA, we also compared *ACE2* expression levels between Asian and non-Asian races in five normal tissues (stomach, thyroid, breast, liver, and pancreas), and did not find a significant difference between them in any tissue (Fig. 1c).

### Association of* ACE2 *expression with immune signatures

**Fig. 2:**
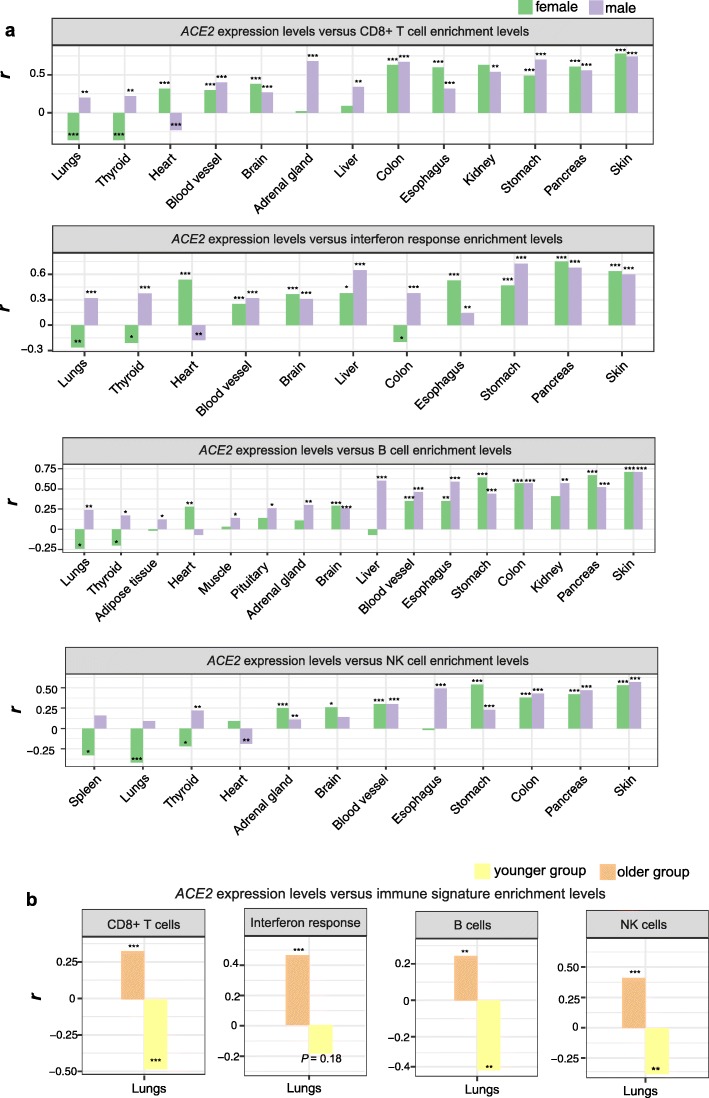
Association of *ACE2* expression with immune signatures in various human tissues. **a** Correlation between *ACE2* expression levels and immune signature enrichment levels in various human tissues in males and females. **b** Correlation between *ACE2* expression levels and immune signature enrichment levels in the lungs and thyroid of older (ages > 49 years) and younger (ages ≤49 years) populations. Pearson’s correlation test was used to calculate the correlation coefficient (*r*) and *P* value in (**a**, **b**). * *P* < 0.05, ** *P* < 0.01, and *** *P* < 0.001. ACE2: Angiotensin-converting enzyme 2; NK: Natural killer

We analyzed the correlations between *ACE2* expression levels and immune signature enrichment levels (CD8+ T cells, interferon response, B cells, and NK cells) in various male and female human tissues. In the skin, digestive system (esophagus, stomach, colon, and pancreas), brain, and blood vessels, we observed significant positive correlations between *ACE2* expression levels and CD8+ T cell enrichment levels in both males and females (Pearson’s correlation test, adjusted *P* < 0.05, 0.27 ≤ *r* ≤ 0.78) (Fig. 2a). In addition, in the thyroid, lungs, adrenal gland, liver, and kidneys, *ACE2* expression levels showed significant positive correlations with CD8+ T cell enrichment levels solely in males (0.20 < *r* < 0.68). However, in the thyroid and lungs, *ACE2* expression levels were negatively correlated with CD8+ T cell enrichment levels in females (*r* = -0.36). In the heart, *ACE2* expression levels had a negative and a positive correlation with CD8+ T cell enrichment levels in males (*r* = -0.23) and females (*r* = 0.32), respectively. Likewise, the interferon response signature had significant positive correlations with *ACE2* expression levels in the skin, digestive system (esophagus, stomach, liver, and pancreas), brain, and blood vessels in both males and females (0.14 ≤ *r* ≤ 0.75) (Fig. 2a). In the thyroid, lungs, kidneys, adrenal gland, colon, and bladder, *ACE2* expression levels had significant positive correlations with the interferon response signature solely in males (0.32 ≤ *r* ≤ 0.82). In contrast, in the thyroid, lungs, and colon, *ACE2* expression levels were negatively correlated with the interferon response signature in females (-0.26 < *r* < -0.20). In the heart, *ACE2* expression levels had a negative and a positive correlation with the interferon response signature in males (*r* = -0.18) and females (*r* = 0.54), respectively. Similar results were observed for B cells and NK cells (Fig. 2a). Collectively, these results demonstrate the commonality and distinction in the association of *ACE2* expression with immune signatures between males and females.

We further analyzed the correlations between *ACE2* expression and immune signatures in younger and older populations. In most individual tissues, the correlations between *ACE2* expression levels and immune signature enrichment levels displayed consistent trends between younger and older populations. However, in the lungs they had a positive and a negative correlation in the older and younger groups, respectively (Fig. 2b). Again, these results suggest the potential difference in the host immune response to coronavirus infection between young and old persons.

## Discussion

We analyzed the expression of *ACE2*, the gene encoding the human host cell receptor of SARS-CoV-2 and SARS-CoV, in 31 normal human tissues. To the best of our knowledge, this is the first study investigating *ACE2* expression across a wide variety of human tissues. We found that although the inflammation of the lungs is the primary symptom of patients with SARS-CoV-2 infection, the lungs had moderate expression of *ACE2* among all tissues. This indicates that SARS-CoV-2 may affect other tissues in addition to the lungs, including male tissues (testis and seminal vesicle). This indication has been confirmed by recent publications [2–4]. For example, Holshue et al. uncovered that stool from a patient infected with SARS-CoV-2 was positive for SARS-CoV-2, suggesting that this virus may infect the gastrointestinal tract [5]. Huang et al. reported the virus-related cardiac injury in five patients with SARS-CoV-2 infection [2]. A study of 99 cases of SARS-CoV-2-related pneumonia revealed the increased susceptibility of males to infection by this virus [3]. In fact, many cases of SARS-CoV-2 have reported symptoms outside of pneumonia, including diarrhea, nausea, vomiting, confusion, headache, and cardiac injury [2–4]. In addition, the predominant symptoms of patients infected with SARS-CoV-2 could be attributed to the fact that the respiratory tract is the readiest transmission approach for the virus.

We found that *ACE2* expression levels showed no significant difference between males and females, between younger and older persons, or between Asian and non-Asian races. This indicates that the infection risk of SARS-CoV-2 and SARS-CoV may have no significant association with sex, age, or race. In fact, like SARS-CoV [14], SARS-CoV-2 can affect males and females equally and can infect young and old persons equally [15]. Nevertheless, the mortality risk for SARS-CoV-2 and SARS-CoV infections appears to be associated significantly with sex and age, with a higher risk for males versus females and old versus young populations [3, 14, 16].

Adaptive and innate immune responses play an important role in fighting off invading coronavirus, even though they may induce a cytokine storm, which is responsible for the immunopathological damage in patients with coronavirus infections [17]. Interestingly, while inflammation of the lungs (pneumonia) is the most common disease caused by coronavirus, the correlations between expression of the ACE2 receptor and immune signatures in the lungs differed between males and females and between young and old persons. This indicates that the host immune response to coronavirus infection could differ between males and females and between young and old persons, partially explaining why males and females and young and old persons have markedly distinct clinical outcomes of coronavirus infections [3, 18]. In addition, it has been demonstrated that lung cells highly expressing ACE2 are more readily infected by SARS-CoV [19]. We found that the correlation between *ACE2 *expression and immune signatures was negative in the lung tissue of females and younger persons, but it was positive in the lung tissue of males and older persons. Collectively, this means that: i) if the lungs highly expressing ACE2 are infected by SARS-CoV-2 in females or young persons, they will have weaker immune signatures, and ii) if the lungs highly expressing ACE2 are infected by SARS-CoV-2 in males or older persons, they will have stronger immune signatures. This suggests that an excessive immune response (cytokine storm or immunopathological damage) could be more likely to occur in males and old persons with SARS-CoV or SARS-CoV-2 infection.

This work provides some basis for our next-step investigation of ACE2 expression at protein level. To understand the potential mechanism of interaction between SARS-CoV2 and ACE2, we will explore the possible enrichment and viability of SARS-CoV2 in different tissues alongside different expression level of ACE2. We will try to clarify the survival, death, transfer, and transformation of SARS-CoV-2 in different tissue niches, and explore the virus in vivo inactivation in human body during and after epidemic. In addition, our pilot data suggest that cigarette smoke or nicotine inhalation inhibits the expression of ACE2/AT2R in multiple tissues, including the brain, heart and lungs, thus disrupting the balance within the renin-angiotensin system.

Our study has several limitations. First, the findings from the bioinformatics analysis need to be further validated by experimental and clinical data. In particular, because mRNA expression pattern of ACE-2 is not necessarily the same as its protein expression pattern across tissues due to some factors probably affecting the mapping from mRNA level to protein level, such as post-translational modification, the validation of our mRNA-based findings at protein level is important and worth further investigation. Second, the tissue mRNA expression did not capture the mRNA expression in individual cell types that would be important for precisely understanding the virus-host interaction. An analysis of the single-cell RNA-Seq data may address this issue. However, because the single-cell RNA-Seq data across such a wide variety of human tissues are lacking, it is not realistic to perform such an analysis. Third, the comparison of *ACE2* expression levels between Asian and non-Asian races in TCGA datasets was performed in a small number of samples, and thus the associated results need to be verified in larger populations. Finally, our data cannot explain the epidemic divergence between SARS-CoV-2 and SARS-CoV in that SARS-CoV-2 is less virulent but more infectious than SARS-CoV. An in-depth analysis of the genomic and protein structures of both coronaviruses would warrant exploring the mechanism underlying the epidemic divergence between SARS-CoV-2 and SARS-CoV.

## Conclusions

ACE2 is expressed in various human tissues in addition to the lungs, indicating that SARS-CoV-2 may infect other tissues aside from the lungs. *ACE2* expression levels have no significant differences between males and females, between young and old persons, and between Asian and non-Asian races in various human tissues, indicating that SARS-CoV-2 may infect persons with different sexes, ages, and races equally. The correlations between *ACE2* expression and immune signatures differ between males and females and between young and old persons in the lungs, indicating that the different host immune responses to SARS-CoV-2 infection may partially explaining why males and females and young and old persons infected with this virus have markedly distinct disease severity. This study provides new insights into the role of ACE2 in the SARS-CoV-2 pandemic and insights into understanding the associations of symptoms, sex, age, and race with SARS-CoV-2 infection.

## Data Availability

The GTEx and TCGA gene expression profiling datasets for human normal tissues were downloaded from the UCSC Xena project (https://xenabrowser.net/datapages/) and the Genomic Data Commons Data Portal (https://portal.gdc.cancer.gov/), respectively.

## Abbreviations

SARS-CoV-2: Severe acute respiratory syndrome coronavirus 2
ACE2: Angiotensin-converting enzyme 2
GTEx: Genotype-tissue expression
TCGA: The cancer genome atlas
HPA: Human protein atlas
NK: Natural killer
